# Screening in General Health Care

**Published:** 2004

**Authors:** Marcia Russell

**Affiliations:** Marcia Russell, Ph.D., is a senior research scientist at the Prevention Research Center, Berkeley, California

The article “Screening for Alcohol Problems” by Stewart and Connors and other articles in this issue and the companion issue of *Alcohol Research & Health* examine in detail how screening can be used in a variety of settings to detect harmful alcohol use. The purpose of this sidebar is to provide a broader view of screening and its role in general health care. Identifying appropriate conditions for screening and developing accurate tools for their diagnosis is an ongoing and important area of research. Here, chronic hepatitis C infection is used as an example of an alcohol-related health problem for which research on screening is urgently needed.

## Brief History of Screens and Preventive Services

Screening tests, together with counseling interventions, immunizations, and chemoprophylactic regimens (i.e., courses of treatment using chemical agents to prevent disease), are all services offered in general health care settings that are designed to prevent a disease or intervene in its early stages.

Screening as a cornerstone of primary health care delivery is a relatively recent medical practice that grew out of public health advances made in the 1930s and 1940s (Berg and Allan 2001). Screening tests and primary preventive advice proliferated in the 1950s and 1960s, a period during which the now classic story of screening newborns for phenylketonuria (PKU) unfolded.

PKU is a genetic abnormality that occurs in about 1 in 12,000 North American births (O’Flynn 1992). Those afflicted are unable to metabolize the essential amino acid phenylalanine, an inability that causes severe mental retardation. If affected infants are identified early and fed a very low protein diet, this retardation can be avoided.

As screening for PKU and other simple screening methods showed their effectiveness in controlling preventable diseases or conditions, the demand for them escalated, which in turn has revealed barriers to providing preventive care. Among these barriers are inadequate reimbursement by health insurance carriers to health professionals for providing preventive services, inconsistent or inadequate health care delivery across a range of care settings, and insufficient time for busy clinicians to provide the range of recommended preventive services to all patients (U.S. Preventive Services Task Force 1996; Yarnall et al. 2003). Even in settings that do not have these problems, health professionals may fail to provide preventive services because they do not know which ones are most effective.

When deciding whether to screen asymptomatic people for disease, the care provider should determine if the potential benefits of identifying and preventing the development of a health problem outweigh the cost and potential harm associated with the screening process, according to the principles of early disease detection published by the World Health Organization (Wilson and Junger 1968). Whitby (1974) modified the principles slightly (see table 1), adding the caveat that treating a disease in the latent or early symptomatic stage should have a favorable effect on outcome.

### The U.S. Preventive Services Task Force

After the publication of the WHO principles, researchers incorporated them into critical scientific reviews of screening procedures (e.g., Russell 1982). In 1984, the U.S. Public Health Service commissioned a 20-member non-Federal panel, the U.S. Preventive Services Task Force (USPSTF), to systematically review the scientific evidence on individual clinical preventive services and to make recommendations to practitioners about what services they should routinely offer (Lawrence and Mickalide 1987). Members of this panel met regularly between 1984 and 1988 and developed recommendations regarding 169 preventive services for 60 topic areas, which they published in 1989 as the *Guide to Clinical Preventive Services*. These recommendations influenced preventive medicine and “accelerated a growing movement to replace traditional ‘expert consensus’ methods for developing clinical recommendations with a systematic and explicit process for reviewing evidence and of linking clinical practice recommendations directly to the quality of the science” (Woolf and Atkins 2001, p. 14).

The second USPSTF was established in 1990 to expand this review to additional topic areas and update recommendations based on new scientific evidence regarding effectiveness (Sox and Woolf 1993). The second edition of the *Guide to Clinical Preventive Services*, published in 1996, assessed mor women, and children. This guide emphasized the importance of:

- Interventions that address patients’ personal health practices
- The need for clinicians and patients to share decisionmaking regarding the use of preventive services
- The need for clinicians to be selective in ordering tests and providing preventive services
- The desirability of delivering preventive services to people with limited access to medical care
- Community-level interventions, which may be more effective than clinical preventive services in addressing some health problems.

In 1998, the Agency for Healthcare Research and Quality (AHRQ) convened the current USPSTF to continue the work of previous panels. Beginning in 2001, this 15-member expert panel began releasing reports summarizing its reviews and recommendations regarding updates of previous assessments or assessments of new topics. (For information concerning the USPSTF’s 2004 recommendation that primary care settings are suitable locations for offering screening and behavioral interventions to reduce alcohol misuse by adults, including pregnant women, see the textbox in the article by Fleming in the companion issue.)

These reports have been published in relevant medical journals and are posted on the AHRQ Web site (www.preventiveservices.ahrq.gov). The work of the panel is supported by outside experts and an evidence-based practice center at Oregon Health and Science University that helps to identify high-priority topics for USPSTF assessment, produces systematic reviews of relevant research on each topic, and works with USPSTF members to draft new chapters of the *Guide to Clinical Preventive Services*. In addition to reviews and recommendations developed by the USPSTF, the AHRQ National Guideline Clearinghouse (www.guideline.gov) provides access to guidelines developed by other entities.

### Guidelines for Evaluating Screening Tests

Over the years, the methods employed to develop evidence-based guidelines for clinical practice have matured. To take full advantage of these advances, the current USPSTF formed a methods subcommittee, the Methods Work Group, to evaluate procedures that were used to develop recommendations and to identify issues for which sound methodology is lacking (Harris et al. 2001). Findings of the work group and discussions with the larger task force led to the formulation of current procedures regarding the scope and selection of topics, review of the evidence, assessment of the net benefits, extrapolation and generalization, translation of evidence into recommendations, drafting of the report, and external review.

To review the evidence, the task force introduced what it called causal pathways to map out specific linkages in the evidence that must be present for a preventive service to be considered effective. A generic causal pathway showing the key questions to be addressed in evaluating a screening test is illustrated in the figure (Harris et al. 2001). More conservative evaluations of screening emphasize important health outcomes, such as morbidity and mortality, rather than intermediate outcomes, which might include changes in physiologic measures or behaviors associated with health risks.

The quality of the evidence supporting each link is evaluated at three levels: the individual study, the linkage, and the entire screening process. Once the task force has evaluated the evidence to support a screening test, it assesses the net benefit, taking into consideration benefits from the individual and population perspectives, and evaluates direct and indirect harms. Although the scientific evidence is of primary importance, when translating evidence into recommendations, the task force also considers other issues such as cost-effectiveness (Saha et al. 2001), resource prioritization, logistical factors, ethical and legal concerns, and patient and societal expectations. The task force assigns letter codes to its recommendations, A through D and I, and employs standard language, as shown in table 2 (Harris et al. 2001).

The I rating of insufficient evidence is a new recommendation category, added to differentiate between clinical preventive services that were previously rated C because there was insufficient evidence to support their inclusion and those rated C because they were associated with small net benefits. This is an important distinction because for some conditions it may not be feasible to conduct the randomized clinical trials needed to provide good-quality scientific evidence for assessing benefits associated with screening, even though substantial benefits might be involved. In such cases, some groups will recommend screening, even though the USPSTF may conclude that data are inadequate to accurately weigh the overall benefits and risks of screening in otherwise healthy asymptomatic adults. An example of this is screening for the hepatitis C virus (HCV).

**Figure:**
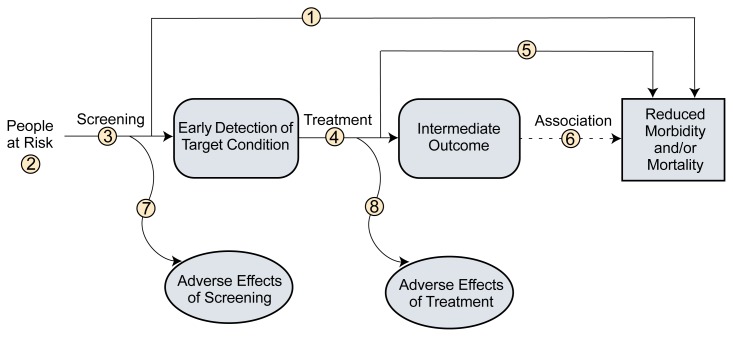
Generic analytic framework for screening topics. Numbers refer to the following key questions: (1) Is there direct evidence that screening reduces morbidity and/or mortality? (2) What is the prevalence of disease in the target group? Can a high-risk group be reliably identified? (3) Can the screening test accurately detect the target condition? What are the sensitivity and specificity of the test? Is there significant variation between examiners in how the test is performed? In actual screening programs, how much earlier are patients identified and treated? (4) Does treatment reduce the incidence of the intermediate outcome? Does treatment work under ideal clinical trial conditions? How do the efficacy and effectiveness of treatments compare in community settings? (5) Does treatment improve health outcomes for people diagnosed clinically? How similar are people diagnosed clinically to those diagnosed by screening? Are there reasons to expect people diagnosed by screening to have even better health outcomes than those diagnosed clinically? (6) Is the intermediate outcome reliably associated with reduced morbidity and/or mortality? (7) Does screening result in adverse effects? Is the test acceptable to patients? What are the potential harms, and how often do they occur? (8) Does treatment result in adverse effects? SOURCE: Harris et al. 2001.

### The Debate Over Screening for HCV

The USPSTF, the Centers for Disease Control and Prevention (CDC), and the National Institutes of Health (NIH) Consensus Panel for the Management of HCV all recommend against routine screening for HCV in asymptomatic people who are not at increased risk for infection (i.e., the general population) (CDC 1998; NIH Consensus Development Program 2002; Chou et al. 2004; USPSTF 2004). This is a grade D recommendation. In addition task force found insufficient evidence to recommend for or against routinely screening for HCV infection in adults at high risk for infection, resulting in a grade I recommendation (Calonge et al. 2004). In contrast, both the NIH Consensus Panel and the CDC do recommend routinely screening people at high risk for hepatitis C infection (Alter et al. 2004), although their definitions of high-risk groups differ.

There are several reasons to screen high-risk populations for chronic hepatitis C infections: to evaluate infected people for antiviral treatment, to immunize them against hepatitis A and B, to counsel them to avoid hepatotoxins—especially alcohol consumption—and to keep them from transmitting HCV to others. Although the pathophysiology of liver disease and clinical experience provide strong support for these interventions, no randomized trials or longitudinal cohorts have compared outcomes between patients in the high-risk populations who were screened and those who were not screened for HCV infection. Such trials would pose ethical and feasibility problems, given the natural history of hepatitis C viral infections.

HCV infection is relatively rare, affecting only 2.3 percent of the adult population (Alter et al. 1999), and the disease may take several decades to develop (Alter and Seeff 2000). Although it accounts for approximately 40 percent of chronic liver disease cases and 10,000 to 12,000 deaths per year, the outcome of infection is quite variable. People with acute HCV infection typically are either asymptomatic or have a mild illness that may go undiagnosed. Chronic HCV develops in 75 to 85 percent of cases, but only about 30 percent of chronic cases progress to severe liver disease (CDC 1998). As discussed by Alter and Seeff (2000), studies of outcomes based on referrals to tertiary care facilities (i.e., hospitals and clinics that have specialists and more sophisticated equipment and technology than found in primary care or general practitioner settings) give an unduly negative picture of outcomes because patients who do not become ill are not represented. In contrast, prospective studies of people infected by HCV have found relatively low rates of cirrhosis, liver cancer, and liver-related mortality. Many of these studies are based on small and/or highly selected samples and have relatively short followup periods of 20 years or less, and thus cannot answer questions about how the disease progresses in more representative samples of the population over the third and fourth decades of infection (Seeff 2002). It also is unknown whether successful antiviral treatment would improve the quality of life for people with chronic hepatitis C infections in whom liver disease does not progress.

HCV screening is associated with substantial costs. Even though laboratory tests for HCV antibodies are highly specific, the false positive rate in asymptomatic general population samples averages 35 percent (CDC 2003). This produces unnecessary anxiety and requires expensive confirmatory testing, both to eliminate false positive findings and to determine whether the infection has resolved or is still active. False positive rates are substantially lower in high-risk, symptomatic populations.

HCV testing also entails risks for the patient. Liver biopsies are needed to evaluate the progression of liver disease to determine whether a patient should receive antiviral treatment. Antiviral treatment itself is expensive, debilitating, and not always successful despite the fact that current antiviral treatment with pegylated interferon and ribavirin is substantially more effective than earlier regimens based on interferon monotherapy (Di Bisceglie and Hoofnagle 2002).

In the case of PKU, the benefits associated with screening and the preventive dietary intervention were so obvious and dramatic that randomized controlled trials never were conducted. However, this is not the case with HCV. Years of rigorously conducted research are needed to fully document the benefits and costs associated with clinical preventive services for chronic hepatitis C infection, and the USPSTF strongly encouraged this investigation. (This is a particularly relevant topic for alcohol researchers; for reviews of alcohol and HCV, see Jamal and Morgan 2003, Morgan et al. 2003, and Peters and Terrault 2002.)

## Conclusion

Screening tests and other interventions for an increasing number of conditions are now included as routine aspects of preventive services offered in general health care settings. As demonstrated by the principles of early disease detection and the methodologies developed by the USPSTF to evaluate the safety and cost-effectiveness of screens, research plays a critical role in determining which preventive services will be adopted and maintained in the future.

## Figures and Tables

**Table 1:** Principles of Early Disease Detection

The condition being sought should be a significant health problem. The natural history of the condition should be understood. There should be a recognizable latent or early symptomatic stage. There should be a screening test or examination capable of detecting the disease in its latent or early symptomatic stage, and the test should be acceptable to the population. There should be an acceptable treatment for people identified as having the disease. Treatment in the latent or early symptomatic stages of the disease should favorably influence its course and prognosis. The facilities to diagnose and treat patients identified in the screening program should be available. There should be an agreed policy on whom to treat as patients. The cost of case-finding, including the cost of diagnosis and treatment, should be reasonable in terms of its relationship to the cost of medical care as a whole. Case-finding should be a continuing process, not a “one-shot” project.

SOURCE: Wilson and Jungner 1968; Whitby 1974.

**Table 2:** U.S. Preventive Services Task Force Recommendations

Code	Definition*
A	The USPSTF strongly recommends that clinicians routinely provide [the service] to eligible patients. (The USPSTF found good evidence that [the service] improves important health outcomes and concludes that benefits substantially outweigh harms.)
B	The USPSTF recommends that clinicians routinely provide [the service] to eligible patients. (The USPSTF found at least fair evidence that [the service] improves important health outcomes and concludes that benefits outweigh harms.)
C	The USPSTF makes no recommendation for or against routine provision of [the service]. (The USPSTF found at least fair evidence that [the service] can improve health outcomes but concludes that the balance of the benefits and harms is too close to justify a general recommendation.)
D	The USPSTF recommends against routinely providing [the service] to asymptomatic patients. (The USPSTF found at least fair evidence that [the service] is ineffective or that harms outweigh benefits.)
I	The USPSTF concludes that the evidence is insufficient to recommend for or against routinely providing [the service]. (Evidence that [the service] is effective is lacking, of poor quality, or conflicting, and the balance of benefits and harms cannot be determined.)

* All statements specify the population for which the recommendation is intended and are followed by a rationale statement providing information about the overall grade of evidence and the net benefit from implementing the service.

SOURCE: Haxrris et al. 2001.
